# The Role of GFAP in Post-Mortem Analysis of Traumatic Brain Injury: A Systematic Review

**DOI:** 10.3390/ijms26010185

**Published:** 2024-12-28

**Authors:** Matteo Antonio Sacco, Saverio Gualtieri, Alessandro Pasquale Tarallo, Maria Cristina Verrina, Jasmine Calafiore, Aurora Princi, Stefano Lombardo, Francesco Ranno, Alessandro Di Cello, Santo Gratteri, Isabella Aquila

**Affiliations:** Institute of Legal Medicine, Department of Medical and Surgical Sciences, Magna Graecia University, 88100 Catanzaro, Italy; matteoantoniosacco@gmail.com (M.A.S.); saverio.gualtieri@studenti.unicz.it (S.G.); alessandropasquale.tarallo@studenti.unicz.it (A.P.T.); mariacristina.verrina@studenti.unicz.it (M.C.V.); jasmine.calafiore@studenti.unicz.it (J.C.); aurora.princi@studenti.unicz.it (A.P.); stefano.lombardo@studenti.unicz.it (S.L.); francesco.ranno@studenti.unicz.it (F.R.); alessandro.dicello@studenti.unicz.it (A.D.C.); gratteri@unicz.it (S.G.)

**Keywords:** traumatic brain injury, GFAP, biomarker, post-mortem, forensic science, astrocyte damage, cerebrospinal fluid

## Abstract

Traumatic brain injuries (TBIs) are a leading cause of mortality and morbidity, particularly in forensic settings where determining the cause of death and timing of injury is critical. Glial fibrillary acidic protein (GFAP), a biomarker specific to astrocytes, has emerged as a valuable tool in post-mortem analyses of TBI. A PRISMA-based literature search included studies examining GFAP in human post-mortem samples such as brain tissue, cerebrospinal fluid (CSF), serum, and urine. The results highlight that GFAP levels correlate with the severity of brain injury, survival interval, and pathological processes such as astrocyte damage and blood–brain barrier disruption. Immunohistochemistry, ELISA, and molecular techniques were commonly employed for GFAP analysis, with notable variability in protocols and thresholds among studies. GFAP demonstrated high diagnostic accuracy in distinguishing TBI-related deaths from other causes, particularly when analyzed in CSF and serum. Furthermore, emerging evidence supports its role in complementing other biomarkers, such as S100B and NFL, to improve diagnostic precision. However, the review also identifies significant methodological heterogeneity and gaps in standardization, which limit the generalizability of findings. Future research should focus on establishing standardized protocols, exploring biomarker combinations, and utilizing advanced molecular tools to enhance the forensic application of GFAP.

## 1. Introduction

### 1.1. Understanding Head Trauma

Head trauma is a complex medical condition that encompasses a broad range of injuries, affecting the scalp, skull, or brain. It is categorized based on the nature and severity of the injury [1]. The two primary types of head trauma are open and closed head injuries. Open head injuries occur when an object penetrates the skull, leading to direct damage to the brain tissue, and account for about 30% of traumatic brain injuries (TBIs) [2]. Closed head injuries, on the other hand, happen when blunt force impacts the skull without breaking it, but still causes significant brain damage. Common causes include falls, motor vehicle accidents, and violent assaults [3].

In the context of post-mortem investigations, understanding the mechanisms and patterns of injury is critical for identifying traumatic brain injury (TBI) as a cause of death. Symptoms observed in living patients, such as confusion or loss of consciousness, cannot be assessed in forensic settings. Therefore, biological and structural evidence gathered during autopsies becomes vital for determining the presence and severity of TBIs. Recognizing specific injury patterns, combined with reliable biomarkers, enhances the ability to confirm TBIs and differentiate them from other causes of death.

### 1.2. Medico-Legal Implications of Head Trauma

Head trauma plays a crucial role in legal cases, often serving as a pivotal factor in determining liability and culpability. Traumatic brain injury (TBI) is a significant cause of disability and mortality, particularly among individuals under the age of 45 [1]. Therefore, its implications in legal settings cannot be understated. The presence of head trauma may influence the outcome of personal injury claims, criminal cases, and even workplace injury disputes. In many instances, head trauma can substantially affect crime scene reconstruction, providing critical insights into the events leading up to the injury [4].

Proving head trauma in forensic contexts presents challenges due to variability in injury presentations and the potential for pre-existing conditions. Imaging techniques, while helpful in living patients, have limited utility in post-mortem settings [5]. Instead, biomarker analysis and histological examinations offer a more definitive approach to understanding brain damage. For example, markers like GFAP can provide objective evidence of TBI, addressing medico-legal complexities and supporting the reconstruction of events leading to death.

### 1.3. Current Diagnostic Methods for Head Trauma

Traditional diagnostic techniques for head trauma have long played a crucial role in medical settings. Tools like CT scans and MRIs can reveal structural damage, aiding immediate clinical decisions [5]. However, these methods have significant limitations when applied to post-mortem investigations. For instance, diffuse axonal injuries and microscopic damage, which are common in TBIs, may not always be visible using standard imaging [6].

Post-mortem investigations rely heavily on autopsy findings, histopathology, and emerging biomarkers to assess brain injuries. Histological techniques, such as staining for astrocytic markers, provide insights into cellular damage and brain responses to trauma. Nevertheless, these methods alone cannot always capture the dynamic molecular changes associated with TBI, underscoring the need for reliable biomarkers.

Recent advancements in diagnostic technology have shown promise in overcoming the limitations of traditional methods. Emerging biomarkers are garnering attention for their potential to serve as reliable indicators of brain damage from TBI. They could potentially fill the diagnostic gap by providing insights into the molecular changes following an injury, thus offering a more comprehensive picture of the patient’s condition. These innovations are paving the way for more accurate and detailed assessments of head trauma, which could revolutionize medico-legal considerations of these injuries.

### 1.4. Need for Reliable Markers in Head Trauma

Biomarkers play a crucial role in the diagnosis and treatment of head trauma, providing essential insights into the extent and nature of brain injuries. The search for potential markers in the realm of head trauma is ongoing, with numerous candidates being researched and developed. The identification of reliable biomarkers has the potential to revolutionize the way TBIs are diagnosed and managed. Recent advancements have highlighted several promising biomarkers that can serve as diagnostic and prognostic tools. These include proteins such as S100B, GFAP, and UCH-L1, which are being evaluated for their sensitivity and specificity in detecting brain injuries [7]. The use of biomarkers during the acute phase of a TBI represents a significant advancement, providing a quicker, less invasive, and potentially more accurate assessment of brain injuries [8]. As research progresses, these biomarkers could become integral components of standard diagnostic protocols, leading to improved patient outcomes and a deeper understanding of the complex pathophysiology of head trauma.

The impact of reliable biomarkers extends beyond clinical settings, influencing both legal and medical outcomes in cases of head trauma. The use of biomarkers such as GFAP also improves the medico-legal integrity of TBI cases. Recent research highlights GFAP’s ability to distinguish traumatic brain injuries from other causes of death by detecting astrocytic damage [9]. Its presence in cerebrospinal fluid (CSF) or serum post-mortem can indicate acute brain injury, making it an essential tool in forensic investigations. Additionally, GFAP’s specificity for astrocytic injury allows for more accurate differentiation between traumatic and non-traumatic brain conditions [7,8,10]. By providing quantifiable evidence, biomarkers address ambiguities in post-mortem analyses, strengthening conclusions in legal contexts. This advancement reduces reliance on subjective assessments and supports the development of standardized protocols for TBI diagnostics in forensic settings.

### 1.5. Understanding GFAP (Glial Fibrillary Acidic Protein)

Glial Fibrillary Acidic Protein (GFAP) is a major intermediate filament protein predominantly found in astrocytes, the star-shaped glial cells in the central nervous system (CNS) [1]. Structurally, GFAP belongs to the type III intermediate filaments family and plays a critical role in maintaining the structural integrity of cells. It consists of a central rod domain, which is crucial for filament assembly, flanked by non-helical head and tail domains that contribute to the protein’s functional properties [1]. The expression of GFAP is tightly regulated at both the transcriptional and post-transcriptional levels, allowing it to adapt to the dynamic needs of the CNS [2].

GFAP serves several vital functions in the central nervous system, primarily through its role in maintaining astrocyte structure and function [3]. It facilitates cell shape and motility, enabling astrocytes to support neuronal cells by maintaining the blood–brain barrier and regulating blood flow [1]. Additionally, GFAP plays a role in cell communication, aiding in the formation of synapses and the propagation of neural signals. It also contributes to the repair and scarring process following CNS injuries, a phenomenon known as astrogliosis [1]. These functions highlight the importance of GFAP in preserving the homeostasis and overall health of the CNS.

GFAP is particularly significant in the context of brain injuries because its expression increases following astrocytic damage, a process known as astrogliosis. This response is commonly observed in cases of traumatic brain injury (TBI), where GFAP serves as an indicator of astrocyte activation and cell injury [3]. In post-mortem analyses, elevated GFAP levels in brain tissues, cerebrospinal fluid (CSF), or serum can serve as a reliable biomarker for identifying TBI [4].

While GFAP has been extensively studied in living patients, its utility in post-mortem settings is increasingly recognized. By detecting and quantifying GFAP, forensic pathologists can assess the presence and severity of brain injuries, even when structural damage is not visually evident. This capability makes GFAP a valuable tool for distinguishing TBIs from other causes of death and understanding the mechanisms underlying fatal head trauma [11,12,13].

The objective of this systematic review is to critically examine and synthesize the scientific evidence regarding the role of glial fibrillary acidic protein (GFAP) in post-mortem investigations of traumatic brain injuries (TBIs). This review specifically focuses on evaluating the utility of GFAP as a biomarker for identifying brain injuries in forensic settings, exploring the methodologies used to detect GFAP in post-mortem samples, and assessing its diagnostic potential. By consolidating current findings, this study aims to address existing gaps in the literature and provide recommendations for future research to enhance the application of GFAP in forensic and medico-legal contexts.

## 2. Materials and Methods

This systematic review was conducted following the guidelines outlined by the PRISMA (Preferred Reporting Items for Systematic Reviews and Meta-Analyses) statement. A comprehensive literature search was performed using the PubMed, Scopus and Web of Science databases, to identify studies examining the role of glial fibrillary acidic protein (GFAP) in post-mortem analyses of traumatic brain injuries (TBIs). The search included articles published up to 10 December 2024 (date of the last update), and the strategy involved using the following key terms: “GFAP AND traumatic brain injury AND autopsy”.

Inclusion criteria were applied to select relevant studies for review. Studies were included if they involved the post-mortem analysis of human subjects, investigated GFAP in the context of TBI, and provided original data using histological, biochemical, or molecular techniques. Exclusion criteria included non-peer-reviewed articles, studies focused solely on animal models, studies including living subjects and those without explicit methodological details. The search process was supplemented by manual screening of reference lists from eligible articles to ensure comprehensive coverage. Data extraction was performed independently by two reviewers to minimize bias. Information was collected on study characteristics, including sample size, type of biological sample analyzed (e.g., brain tissue, cerebrospinal fluid, or blood), type of trauma, methods of GFAP analysis, and main findings. Discrepancies between reviewers were resolved through discussion or consultation with a third reviewer.

The quality of the included studies was evaluated using the Newcastle-Ottawa Scale (NOS), a standardized tool widely used for observational studies. The NOS focuses on three main domains: the selection of study participants, the comparability of groups, and the clarity of outcome assessment and reporting. This evaluation revealed that most of the studies demonstrated adequate methodological rigor; however, there was some variability in sample sizes and reporting consistency across the papers.

**Figure 1:**
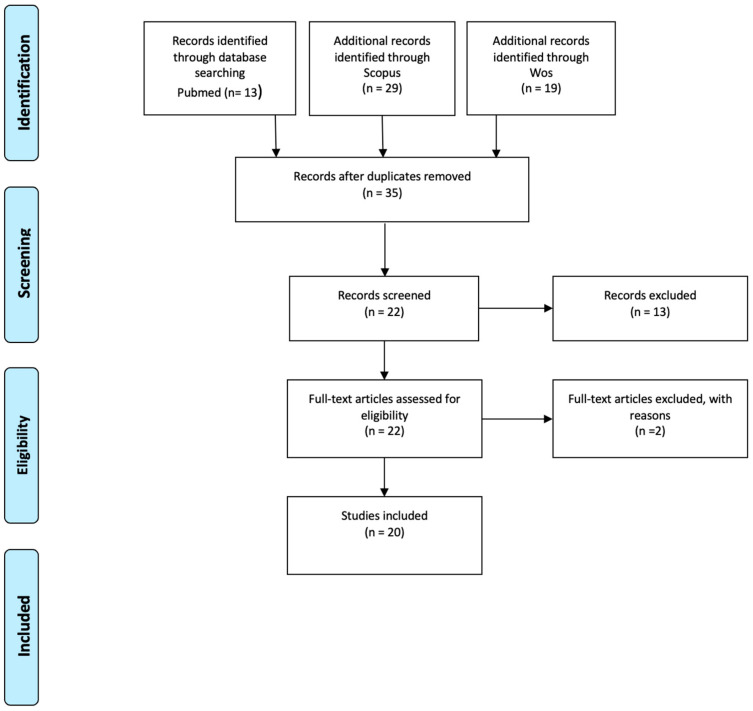
Searching method using PRISMA flowchart.

From the analysis of the keywords, 35 papers emerged. After reading the abstracts, 22 papers were selected for full-text reading. Furthermore, 20 papers met the inclusion criteria and were therefore selected (Figure 1).

## 3. Results

### 3.1. Analysis of the Emerging Studies

The analysis of the included studies highlights the critical role of glial fibrillary acidic protein (GFAP) as a biomarker in post-mortem traumatic brain injuries (TBI). A clear temporal trend emerges regarding brain alterations and GFAP expression after trauma. For instance, Oehmichen et al. (2003) documented predictable histomorphological changes, such as axonal swelling and mesenchymal proliferation, which vary depending on the post-trauma survival interval, aiding in estimating the time since trauma [14]. Similarly, Duncea-Borca et al. (2018) demonstrated increased GFAP density in injured areas, correlating it with glial scar formation within 1–2 months post-trauma [15]. The relationship between GFAP and injury severity was further analyzed. Li et al. (2012) highlighted GFAP and S100 protein immunopositivity as useful indicators of damage severity and pathological responses [16]. Goede et al. (2015) emphasized GFAP’s role in identifying blood–brain barrier (BBB) damage and cortical vessel rupture in pericontusional zones [17].

Chirica et al. (2017) explored GFAP alongside S100B and NSE, suggesting their utility in cerebrospinal fluid (CSF) and blood for identifying contusions and diffuse axonal injuries [15]. These findings underscore GFAP’s diagnostic relevance in assessing trauma dynamics. Similarly, Wang et al. (2012) confirmed GFAP’s utility, alongside proteins like bFGF and ssDNA, while Zwirner et al. (2021) demonstrated its accuracy in distinguishing fatal TBI cases when combined with IL-6 [18,19].

Studies have also emphasized astrocytic morphological changes. Sakai et al. (2013) described clasmatodendrosis, associated with acute brain edema and reduced survival time [20]. Meanwhile, Becerra-Hernández et al. (2022) highlighted GFAP overexpression alongside CRYAB in cortical tissues, marking its utility in subacute injuries and reactive astrogliosis [21].

In CSF-based studies, Olczak et al. (2017, 2018) reported significantly elevated GFAP levels in fatal TBI cases, with Dereli et al. (2022) further demonstrating GFAP elevations correlating with astrocytic endfeet damage [22,23,24]. For forensic purposes, Ondruschka et al. (2018) confirmed GFAP’s utility in differentiating TBI-related deaths, while Breitling et al. (2018) noted correlations between GFAP levels and agonal state duration [25]. Postupna et al. (2021) extended GFAP’s relevance to long-term outcomes, linking its expression to early astrocytic activity and potential neurodegeneration [26]. Recent findings by Olczak et al. (2023) highlighted GFAP’s presence in post-mortem serum and urine, demonstrating its reliability as a forensic marker [27].

Collectively, these studies validate GFAP as a robust biomarker for post-mortem TBI analysis, with applications spanning trauma chronology, severity assessment, and forensic investigations.

### 3.2. Analysis of Methodologies

The studies utilized a range of methodologies to detect and analyze GFAP, reflecting its versatility as a biomarker in post-mortem brain injury investigations.

Immunohistochemistry was one of the most frequently employed techniques for localizing GFAP in brain tissues. For example, Olczak et al. (2020) and Oehmichen et al. (2003) combined immunohistochemistry with histological staining to identify trauma-related histomorphological changes, while Sakai et al. (2013) integrated immunofluorescence and confocal microscopy to examine astrocytic alterations such as clasmatodendrosis [14,20,28]. Similarly, Cawsey et al. (2015) used immunohistochemistry to analyze GFAP-positive ependymal cells, correlating their increase with BBB dysfunction and brain edema [29].

For body fluid analysis, ELISA was widely adopted to quantify GFAP levels. Olczak et al. (2018) and Ondruschka et al. (2018) applied ELISA and multiplex assays to cerebrospinal fluid, establishing diagnostic thresholds for distinguishing TBI-related deaths [23,30].

Advanced molecular techniques such as mass spectrometry and qPCR offered deeper insights into brain injury processes. Postupna et al. (2021) combined mass spectrometry with immunohistological assays to analyze astrocytic activation and inflammatory markers [26]. Similarly, Staffa et al. (2012) employed qPCR to study GFAP gene expression and its role in TBI pathology [31].

In summary, immunohistochemistry remains central for GFAP localization in tissue samples, while ELISA and molecular assays provide quantitative data in body fluids and gene expression. These methodologies collectively enhance the understanding of GFAP’s role in post-mortem TBI diagnostics (Table 1).

## 4. Discussion

### 4.1. GFAP as a Biomarker for Post-Mortem TBI Analysis

This systematic review highlights the role of glial fibrillary acidic protein (GFAP) as a significant biomarker in the post-mortem analysis of traumatic brain injury (TBI). GFAP has consistently demonstrated its reliability as an indicator of brain injury severity, with elevated levels observed in patients with severe TBI, making it a potential tool for assessing the extent of brain trauma [33]. As an astrocytic protein released into the bloodstream following neuronal damage, GFAP provides a measurable marker that reflects the severity of brain injury [34]. Studies have shown a clear correlation between increased GFAP levels and prolonged agony times in TBI patients, underscoring its importance in indicating injury severity. This suggests that GFAP measurement can offer valuable insights into the severity of TBI and may help guide treatment strategies.

Research has also confirmed GFAP’s utility in predicting TBI outcomes. Significant increases in GFAP density several days post-trauma suggests a delayed astrocytic response [15]. GFAP is a useful biomarker for determining “wound age” (time elapsed since trauma) in autopsies [17]. Longitudinal studies tracking GFAP levels over extended periods, such as 21 days post-trauma, reveal a strong association between GFAP levels, increased intracranial pressure (ICP), and overall injury severity [25]. These findings emphasize GFAP as a reliable biomarker for predicting neurological outcomes. Additionally, GFAP’s ability to distinguish TBI cases from controls, particularly in cerebrospinal fluid (CSF) and serum, further supports its role as a diagnostic tool [30,34]. Collectively, these studies highlight the clinical value of GFAP in predicting patient outcomes, allowing for more informed decisions in TBI management. The utility of GFAP as a prognostic marker extends beyond clinical settings to forensic science, where elevated GFAP levels in serum have been recognized as predictors of severe head trauma with important implications for prognosis and treatment planning. In forensic investigations, GFAP provides valuable insights into the severity and timing of brain injuries, aiding in reconstructing injury events and supporting legal proceedings. When used in conjunction with other biomarkers, such as S100B, GFAP enhances diagnostic accuracy, helping healthcare professionals tailor treatment strategies more effectively [15,35]. These consistent findings underscore GFAP’s potential as a cornerstone biomarker in both forensic and clinical evaluation of TBI. In forensic contexts, GFAP has proven effective in distinguishing between cerebral and non-cerebral causes of death [29].

### 4.2. Methodological Considerations

The post-mortem analysis of GFAP involves critical steps to ensure accuracy and reproducibility, including proper sample collection and preparation. Brain tissue and blood samples are typically collected shortly after death to minimize degradation, ensuring precise correlations between biomarkers and clinical features [36]. The brain tissue is often fixed in formalin to preserve its structural integrity before sectioning for analysis, while plasma samples undergo centrifugation to separate blood cells from plasma, where GFAP levels can be quantified. Staining and visualization techniques, such as immunohistochemistry and immunofluorescence, are commonly used to detect and quantify GFAP in brain tissue. These methods rely on specific antibodies that bind to GFAP, enabling its visualization under a microscope. Immunohistochemistry, for instance, reveals GFAP’s presence and distribution within astrocytes, providing valuable insights into the extent of astrogliosis [19,21,37]. Advanced imaging techniques like confocal microscopy enhance the ability to discern subtle GFAP staining patterns, which is crucial for understanding pathological changes in GFAP expression in various neurological conditions.

Quantitative methods such as enzyme-linked immunosorbent assay (ELISA) and Western blotting are essential in measuring GFAP levels in post-mortem samples. ELISA, widely used for measuring GFAP in CSF and plasma, offers high sensitivity and can detect even low levels of GFAP, providing a clear indication of brain injury severity [22,24,38]. Western blotting further complements this by offering insights into GFAP and its degradation products, helping to elucidate the molecular changes associated with neurotrauma. However, post-mortem GFAP analysis faces challenges. Staining artifacts are a significant issue, as neuronal immunopositivity for GFAP can often be attributed to technical errors rather than genuine expression, complicating result interpretation. Additionally, the timing of post-mortem sample collection influences GFAP levels, with prolonged agony times leading to artificially elevated concentrations that may skew results [33]. These factors, along with variability in tissue preservation and the lack of standardized protocols, emphasize the need for refinement in post-mortem GFAP methodologies.

The findings from this review suggest several recommendations for improving diagnostic and treatment strategies for TBI. Incorporating GFAP measurements into routine diagnostic protocols could improve the accuracy of TBI severity evaluations, enabling more personalized treatment plans. Moreover, recognizing GFAP as a specific marker in serum indicates its potential to monitor TBI progression in living patients, offering valuable guidance for therapeutic interventions [14]. It is also crucial to standardize laboratory protocols, particularly in relation to staining techniques, to minimize the risk of staining artifacts and ensure accurate readings [16]. These recommendations can lead to more effective TBI management, improving patient outcomes and informing forensic investigations.

### 4.3. Clinical and Forensic Implications

GFAP’s potential as a biomarker in TBI not only has clinical applications but also opens promising directions for future research. Investigating GFAP alongside other biomarkers, such as P-tau, could improve predictive models for TBI outcomes, as studies have demonstrated that combining biomarkers increases predictive accuracy [18]. Further exploration of the molecular mechanisms underlying GFAP expression and its interaction with neuroinflammatory pathways may provide deeper insights into TBI pathophysiology. Future research should also focus on addressing the gaps identified in post-mortem studies, particularly regarding reactive astrocytes and their role in neurotrauma recovery [31]. These areas of research hold promise for developing innovative strategies to mitigate the impact of TBI and promote recovery.

Furthermore, post-mortem GFAP analysis holds promise in the study of developmental brain disorders. GFAP expression is tightly regulated during brain development and varies across different neurological diseases [2]. Investigating the distinct GFAP isoforms in various cell types could provide specific insights into developmental abnormalities and their progression [39]. Moreover, post-mortem studies focusing on reactive astrocytes have revealed gaps in understanding developmental brain disorders, which suggests promising areas for further research [14]. These research directions are essential for identifying early biomarkers and therapeutic targets for developmental brain disorders, which could lead to improved treatment outcomes.

GFAP levels have also been shown to be valuable in identifying Alzheimer’s disease (AD) [40]. Elevated GFAP levels in the blood are frequently observed in AD patients, suggesting a link between GFAP and the pathological processes of the disease [18]. Notably, individuals with amyloid-β (Aβ) positivity, a hallmark of AD, exhibit higher GFAP levels compared to those without such pathology [18]. This finding underscores GFAP’s potential as a diagnostic marker, aiding in early detection and monitoring of AD progression. Furthermore, studies indicate that AD patients synthesize GFAP at levels significantly higher than controls, further supporting its role in the disease’s pathology [31].

In the context of traumatic brain injury (TBI) and stroke, GFAP has emerged as a key biomarker indicating astrocytic damage and reactivity [30]. Elevated serum GFAP levels are commonly found in patients following an acute stroke or TBI, reflecting the extent of astroglial injury [11]. GFAP’s presence in the blood serves as an indicator of intracerebral hemorrhage, allowing for timely and accurate diagnosis [11]. Post-mortem studies also show a strong correlation between serum GFAP levels and the severity of brain injury, highlighting its diagnostic utility in assessing TBI and stroke outcomes [16]. This makes GFAP a critical tool in both clinical and research settings for understanding and managing brain injuries.

Additionally, GFAP plays a significant role in psychiatric disorders, where its levels often change [41]. For example, patients with major depressive disorder (MDD) exhibit distinct GFAP profiles, which may aid in the differential diagnosis of psychiatric conditions [41]. Research has demonstrated that serum GFAP levels correlate with depression severity, offering a potential biomarker for evaluating treatment efficacy and disease progression [23]. Post-mortem studies have also revealed decreased GFAP levels in brain regions such as the hippocampus, amygdala, and cerebellum in patients with stress-related disorders, further emphasizing its relevance in psychiatric pathology [24]. This evidence supports the potential of GFAP in understanding and diagnosing various psychiatric conditions.

### 4.4. Challenges in Post-Mortem Analysis

The limitations of current GFAP post-mortem analysis methods are substantial. One significant challenge is the degradation of samples due to post-mortem phenomena [30], which can obscure the accurate detection and measurement of GFAP. Biochemical methods are often insufficient for estimating agonal periods accurately, which further complicates the analysis [26]. The reliance on traditional histological and immunohistochemical techniques introduces artefacts that may misrepresent the true condition of neural tissues [42], emphasizing the need for more reliable and precise methods to overcome these challenges.

Technological advancements in GFAP detection are paving the way for more accurate and reliable post-mortem analyses. Recent studies have highlighted the potential of advanced imaging and molecular techniques to better detect astrocyte reactivity, particularly in the context of Alzheimer’s disease [5]. More sophisticated immunohistochemical methods are reducing the risk of staining artefacts [12], while post-translational modification analyses offer deeper insights into the functional state of GFAP in various neurological conditions [1]. These innovations are not only improving the accuracy of post-mortem analyses but also expanding our understanding of GFAP’s role in central nervous system injuries.

The growing recognition of personalized medicine and targeted therapies through post-mortem GFAP analysis is also noteworthy. By examining specific patterns of GFAP expression and its post-translational modifications, researchers can identify biomarkers that may inform personalized treatment regimens [27]. This approach complements the broader trend in genomic research, aiming to leverage biomarker-driven therapies for optimal therapeutic outcomes [43]. Clinical laboratories play a vital role in enabling precise monitoring strategies and developing personalized treatment plans based on post-mortem findings [25,30,33,34,35,36,37,38,40,41,44]. These insights could revolutionize the management of neurological diseases, offering more effective and tailored therapeutic options for patients.

### 4.5. Bias Evaluation

In evaluating the included studies, potential sources of bias were carefully considered, particularly given the relatively small number of studies on this topic. Geographic and demographic variability emerged as a notable factor. The majority of studies were conducted in specific regions, often in Europe and North America, which may limit the generalizability of findings to other populations with different healthcare systems, trauma responses, or forensic protocols. Additionally, variability in patient populations, including differences in age, cause of trauma, and post-mortem intervals, may have influenced GFAP expression levels. Methodological heterogeneity, such as differences in sample collection, tissue preservation, and analysis techniques, also posed a potential risk of bias. While most studies demonstrated methodological rigor, the lack of standardized protocols makes it difficult to compare results directly across studies. Future studies would benefit from addressing these factors to enhance the reliability and generalizability of GFAP findings in post-mortem TBI analysis.

### 4.6. Limitations of the Study

This systematic review has several limitations that must be acknowledged. First, the number of available studies on post-mortem GFAP analysis remains limited, which constrains the scope and generalizability of the findings. Second, significant heterogeneity was observed in the methodologies used, including variations in sample collection timing, preservation techniques, and GFAP quantification methods (e.g., immunohistochemistry, ELISA, and Western blotting). These inconsistencies make it challenging to draw direct comparisons or perform a meta-analysis. Third, most of the studies included small sample sizes, which increases the risk of type II errors and limits the statistical power of the results. Lastly, the geographic concentration of the studies may introduce regional biases, and findings may not fully represent global forensic and clinical settings. These limitations highlight the need for further research to validate and expand on the current findings.

### 4.7. Suggestions for Future Research

Future research should aim to address the limitations identified in this review. Firstly, studies with larger and more diverse patient populations are needed to improve the generalizability of GFAP findings across different geographic, demographic, and clinical contexts. Standardized protocols for sample collection, tissue preservation, and GFAP quantification should be developed to reduce methodological variability and allow for more consistent comparisons between studies. Additionally, longitudinal studies that track GFAP expression across varying post-mortem intervals would provide greater insight into its temporal dynamics and its relationship with survival times.

Another promising direction involves combining GFAP with other biomarkers, such as S100B, neurofilament light (NFL), and tau proteins, to improve diagnostic accuracy and predictive models for brain injury. Advanced molecular techniques, including proteomics and transcriptomics, should be explored to deepen the understanding of GFAP’s role in neuroinflammatory processes and astrocyte reactivity. Finally, future studies should consider the utility of GFAP in other neurological conditions, such as neurodegenerative diseases and psychiatric disorders, to expand its applications beyond TBI. These efforts will contribute to establishing GFAP as a reliable and versatile biomarker in both clinical and forensic settings (Table 2).

## 5. Conclusions

In conclusion, this review underscores the potential of GFAP as a biomarker in post-mortem analyses of TBI and other neurological conditions. Despite the challenges and limitations in current methodologies, ongoing research will refine these techniques and further elucidate GFAP’s role in clinical and forensic applications. Continued investigation into GFAP will be essential for advancing our understanding and management of traumatic brain injuries and related neurological disorders.

## Figures and Tables

**Table 1:** Results of systematic review of the literature.

Authors	Number of Cases	Biological Sample	Type of Trauma	Analysis Method	Results
Oehmichen M., et al., 2003 [14]	305	- Brain Tissue.	- Cortical hemorrhages	- Sandard histological stains - Immunohistochemistry (GFAP) - Analysis of frozen sections.	- Study results show that histomorphological changes in brain tissue samples follow a predictable time course after TBI. - Eighteen major histological criteria were identified, including blood cell reactions, neuronal damage and axonal swelling, astrocytic reaction and mesenchymal proliferation. - The statistical analysis highlighted that the frequency and intensity of each alteration vary based on the post-traumatic survival interval, allowing the time elapsed since the trauma to be estimated.
Oehmichen et al., 2009 [32]	Not specified	- Brain tissue.	- Gunshot wounds to the head, formation of a temporary cavity around the bullet track, with extensive mechanical damage to axons and neurons.	- Histology (GFAP, b-APP, CD68). - CT and MRI.	- GFAP was used to characterize astrocytic damage and establish the extent and timing of traumatic brain injuries. - The combined use of imaging and histological analyses enables the reconstruction of the bullet path, cause of death, and survival time.
Li DR et al., 2012 [16]	168	- Cerebral white matter. - CA4 region of the hippocampus.	- Acute injuries and with delayed death. - Progressive brain dysfunction. - Fatal post-trauma complications.	- Immunocytochemistry (GFAP and protein S100) - Quantitative analysis of GFAP and S100.	- The results suggest that the presence and quantity of astrocytes immunopositive for GFAP and S100 can provide important indications on the severity of brain damage, death dynamics and pathological responses following TBI -Immunopositivity for GFAP and S100 has proven useful in elucidating the cause and process of deaths due to brain injury.
Wang Q et al., 2012 [18]	174	- Parietal lobe. - Hippocampus.	- Cerebral laceration. - Subarachnoid Hemorrhage (SAH). - Intracranial hematoma.	- Immunocytochemical study on bFGF, GFAP, ssDNA.	- These findings provide an in-depth understanding of how different proteins react following traumatic brain injury, offering potential biomarkers to evaluate brain damage and to guide therapeutic strategies in the early stages after trauma.
Staffa K et al., 2012 [31]	12	- Site of the brain lesion. - Area contralateral to the lesion. - Hippocampus. - Cerebellum portion.	- Nine out of twelve road accidents. - Three out of twelve fall-related injuries. - Skull fractures, cerebral contusions, intracranial hemorrhages.	- RNA extraction using Trifast reagent. - Reverse Transcription - qPCR. - Primer design. - pH measurement of injured tissues. - Data analysis using LinRegPCR program.	- The results indicate that the cerebellum reacts quickly to brain trauma and suggest that it may be a key region for further studies on the impact of brain injuries and potential therapies. - Activation of GFAP generally occurred 2–4 days post-trauma, suggesting a delayed astroglial response to damage. - Other genes such as TrkB and Caspase-3 are overexpressed immediately following these injuries.
Sakai K et al., 2013 [20]	36	- Samples of injured cerebral cortex.	- Traumatic subarachnoid hemorrhages. - Chronic and acute subdural hematomas. - Acute epidural hematomas.	- Light microscopy. - Immunohistochemical staining IHC to detect GFAP. - Immunofluorescent staining for p62-K48, p62-K63, and GFAP-K48. - Laser scanning confocal microscope.	- The study results highlighted several key aspects regarding clasmatodendrosis, a morphological change in astrocytes following head trauma. - Cases with clasmatodendrosis showed a significantly shorter median survival time (12 h), and these cases also exhibited a higher frequency of edema and activation of protein degradation pathways. - Astrocyte alterations could affect microvascular functions in the brain, such as the regulation of the blood–brain barrier and the control of cerebral blood flow, especially in the acute phase of head trauma.
Goede A et al. 2015 [17]	42 cases, 13 controls	Samples from - the pericontusional area; - contralateral cortex; - hippocampus -cerebellum.	- Blood vessel rupture -Blood–brain barrier (BBB) damage -Loss of basal membrane integrity -Neuronal death and axonal damage -Reactive astrogliosis.	- Western Blot(NSE and GFAP)	- Significant increase in GFAP after 4 days, suggesting a delayed astrocytic response. - NSE and GFAP are useful biomarkers for determining “wound age” (time elapsed since trauma) in autopsies.
Cawsey et al. 2015 [29]	27 traumatic cases, 14 controls	Human spinal cord	- Skull and/or spinal fractures. - Subarachnoid and subdural hemorrhages. - Brain and spinal cord contusions. - Diffuse axonal injury (DAI). - Cerebral and spinal edema. - Tissue lacerations and necrosis.	- Immunohistochemistry (GFAP, nestin). - Morphometric analysis.	- Increase in GFAP- and nestin-positive ependymal cells after CNS trauma. - Indicates that astrocyte activation is a distinct response from the increase in progenitor cells.
Olczak M et al., 2017 [22]	38	- Blood (serum). - Cerebrospinal fluid from suboccipital puncture. - Frontal cortex tissue.	- Twenty-one out of thirty-eight fatal head injuries. - Seventeen out of thirty-eight deaths from cardiopulmonary failure (control group).	- ELISA. - Histological examination with hematoxylin and eosin. - Immunohistochemistry (anti-Tau, GFAP, CD34, CD68). - Microscopy	- In the group with head trauma, marked clasmatodendrosis of astrocytes was observed, which implies damage to these cells, and damage to astrocyte endfeet, which are crucial for maintaining the blood–brain barrier. These damages were detected through immunostaining with GFAP, indicating a direct impact of trauma on the structure and function of astrocytes and on the components of the blood–brain barrier.
Chirica et al., 2017 [15]	Not specified	- biofluids (CSF, blood) - brain tissue	- Closed traumatic injuries (contusions, intracranial hematomas, and cerebral swelling) - open injuries - intracranial hemorrhage - hydrocephalus, - diffuse axonal injuries	- Western Blot (GFAP, NSE, S100B, UCH-L1)	- S100B and NSE indicated as useful markers for traumatic and ischemic brain injuries - GFAP is relevant in acute and subacute phases to predict neurological prognosis.
Olczak M et al., 2018 [23]	38	- cerebrospinal fluid (CSF) from suboccipital puncture - frontal cortex tissue	- 21 out of 38 fatal head injuries - 17 out of 38 deaths from cardiopulmonary failure (control group)	- ELISA to measure GFAP, NFL, and MBP in CSF - immunohistochemistry for brain tissues	- elevated levels of specific proteins in cerebrospinal fluid (CSF) are correlated with traumatic brain injuries - protein analysis in CSF can be a useful diagnostic tool to determine the extent of traumatic brain injuries in post-mortem studies, complementing other diagnostic techniques such as neurological examination and imaging studies.
Breitling B et al., 2018 [25]	129	- blood serum collected from the right heart (valid samples: 125 out of 129)	- 12 TBIs (4 road accidents, 3 falls, 2 blunt traumas, 1 firearm injury, 1 railway accident) - 3 subarachnoid hemorrhages due to aneurysm - 2 intracerebral hemorrhages - 1 subdural hematoma - 111 non-cerebral primary causes of death	- ELISA	- although GFAP is a useful biomarker for assessing the extent of brain damage in acute conditions such as stroke, its levels in post-mortem samples do not specifically discriminate between cerebral and non-cerebral causes of death. However, its association with the duration of agony provides an interesting perspective for further research on the dynamics of brain damage in the perimortem phase.
Ondruschka B et al., 2018 [30]	84	- CSF - serum	- 42 out of 84 fatal TBIs - 42 out of 84 non-TBI controls	- quantitative multiplex chemiluminescent immunoassays for GFAP, BDNF, NGAL - hemolysis index for post-mortem studies	- in CSF, GFAP proved to be an effective marker for identifying TBI cases. Additionally, in serum, only GFAP was significantly elevated in TBI cases compared to controls. - threshold values for GFAP, BDNF, and NGAL were determined, which could help distinguish deaths caused by TBI from other types of death.
Duncea-Borca RM et al., 2018 [15]	22 cases (13 trauma, 9 controls)	Brain tissue	- Cortical lesions. - Necrosis. - Subdural/subarachnoid hemorrhages. - Astrogliosis.	- Histology and immunohistochemistry (GFAP). - quantitative analysis of GFAP. - positive cell density in lesioned and perilesional areas.	- GFAP density increases with time post-trauma.-Glial scar visible after 1–2 months.-GFAP is useful for estimating the time elapsed since trauma.
Olczak M et al., 2020 [28]	15 cases, 15 controls	- Samples from the frontal lobe (including cingulate cortex and corpus callosum) - One cerebellar hemisphere.	- Severe cranial trauma with focal brain injuries such as contusions, subdural and intraventricular hemorrhages. - Focal hypoperfusion in affected areas. - Concomitant dysfunction in the formation of neurovascular units of the blood–brain barrier (BBB).	- Immunohistochemistry. - Mallory staining.	- The accumulation of plasma proteins in neurons and Bergmann glia confirms BBB dysfunction in the early stages of cranial trauma.
Postupna N et al., 2021 [26]	532	- FFPE brain tissue - Frozen brain tissue	- A total of 107 TBI with loss of consciousness (TBI w/LOC). - A total of 425 not affected by TBI.	- Immunohistochemistry. - Histelide immunoassay. - Luminex assay. - Mass spectrometry. - RNA sequencing. - Neuropathological evaluation.	- The neuropathological effects of traumatic brain injury were examined, showing no significant differences in pathological and inflammatory markers, a low incidence of chronic traumatic encephalopathy (CTE), and minimal changes in gene expression. However, an increase in Tau was observed in the hippocampus. - GFAP was measured to assess the extent of tissue reaction to trauma and the level of brain inflammation, helping to understand how TBI affects long-term brain health and whether there is an association between TBI and increased astroglial activity, which could be an early indicator of future neurodegenerative diseases.
Zwirner J et al., 2021 [19]	100 cases (30 fatal TBI, 70 controls)	CSF and blood	- Acute fatal traumatic brain injury (TBI). - Subdural/subarachnoid hemorrhages. - Neuronal necrosis. - Blood–brain barrier (BBB) dysfunction.	- Quantitative immunoassays for CNS biomarkers: GFAP, NSE, S100B, BDNF. - Acute-phase proteins: IL-6, NGAL, ferritin, LDH.	- Combination of GFAP + IL-6 is highly accurate for diagnosing fatal TBI.
Becerra-Hernández et al., 2022 [21]	10 cases, 3 controls	Contused cortical tissue from frontal and temporal areas	- Severe cranial trauma from road accidents- gunshot wounds-sharp force injuries with brain contusions. - Subdural and intraventricular hemorrhages. - Focal necrosis in affected areas. - Focal hypoperfusion and BBB damage.	- Immunohistochemistry (GFAP and CRYAB) - Immunofluorescence	- Overexpression of GFAP associated with CRYAB in contused tissues is indicative of reactive astrogliosis and has potential as a marker for subacute injuries. - Selective vulnerability of pyramidal neurons is observed during trauma.
Dereli AK et al., 2022 [24]	44 cases (17 fatal trauma, 9 non-fatal trauma, 18 controls)	CSF and blood	- Cranial trauma with skull fractures. - Subarachnoid hemorrhages. - Brain contusions. - Secondary ischemia and hypoxia.	ELISA for GFAP and UCH-L1	- GFAP and UCH-L1 levels not significantly different between groups. - Higher GFAP levels in CSF compared to serum across all groups. - UCH-L1 significantly higher in CSF compared to serum in non-fatal trauma and controls.
Olczak M et al., 2023 [27]	60	- Serum from femoral venous sampling.. - Urine from suprapubic bladder sampling	- Forty out of sixty cases of fatal severe head injuries. - Twenty out of sixty cases of sudden death without signs of head injuries.	- ELISA (double sandwich kit used for GFAP). - statistical analysis of ELISA data using Statistica 13.1 PL software and Microsoft Office Excel 2010.	- The results showed a significant increase in GFAP concentration in serum and urine in study cases compared to control cases, demonstrating GFAP as a biomarker for the post-mortem diagnosis of traumatic brain injuries, given the correlation between elevated GFAP levels and astrocyte damage in the context of head trauma.

**Table 2:** Summary of biological aspects of GFAP.

Aspect	Description
Biological Role	Astrocytic protein released into the bloodstream following brain injury.
Clinical Relevance	Indicator of brain injury severity and progression in TBI patients.
Diagnostic Utility	Distinguishes TBI cases from controls, particularly in CSF and serum.
Prognostic Value	Strong correlation with increased intracranial pressure and neurological outcomes.
Forensic Applications	Assists in reconstructing injury events and supports forensic investigations.
Quantitative Analysis Methods	ELISA and Western blotting are common techniques for measuring GFAP levels.
Challenges in Analysis	Sample degradation, staining artifacts, and variability in tissue preservation complicate results.
Associated Biomarkers	Used alongside S100B and UCH-L1 to improve diagnostic accuracy and prognostic modeling.
Neurological Disorders	Elevated GFAP levels found in Alzheimer’s disease and other neurodegenerative conditions.
Forensic Science Utility	Helps determine the severity and timing of brain injuries in legal and forensic settings.
Future Research Directions	Investigating GFAP isoforms, its interaction with neuroinflammation, and biomarkers combination.
